# An investigation of Australian osteopaths’ attitudes, skills and utilisation of evidence-based practice: a national cross-sectional survey

**DOI:** 10.1186/s12913-019-4329-1

**Published:** 2019-07-17

**Authors:** Matthew J. Leach, Tobias Sundberg, Gary Fryer, Philip Austin, Oliver P. Thomson, Jon Adams

**Affiliations:** 1 Department of Rural Health, University of South Australia, Adelaide, Australia; 2 Australian Research Centre in Complementary and Integrative Medicine (ARCCIM), Faculty of Health, University of Technology Sydney, Sydney, NSW Australia; 3 Musculoskeletal and Sports Injury Epidemiology Center (MUSIC), Institute of Environmental Medicine, Karolinska Institutet, Stockholm, Sweden; 4 College of Health & Biomedicine, Victoria University, Melbourne, Victoria Australia; 5 Department of Pain Management, Greenwich Hospital, Sydney, Australia; 6 University College of Osteopathy, London, United Kingdom; 7 Clinical-based Human Research Department, Centre for Osteopathic Medicine Collaboration (COME), Pescara, Italy

**Keywords:** Attitude, Barriers, Enablers, Evidence-based practice, Osteopathy, Skill, Survey, Training

## Abstract

**Background:**

Osteopaths are an integral member of the health care team, playing a pivotal role in the provision of care for patients with musculoskeletal disorders. Osteopaths, like other health care providers, are under increasing pressure to deliver evidence-based health care and to improve patient outcomes. However, the extent to which osteopaths engage in evidence-based practice (EBP), particularly in Australia, is not well understood. This study therefore set out to investigate the attitudes, skills and use of EBP, and perceived barriers and enablers of EBP uptake, among osteopaths practicing in Australia.

**Methods:**

National cross-sectional survey of Australian registered osteopaths. Eligible participants were invited by email and other digital media recruitment strategies to complete the online Evidence-Based Practice Attitude and Utilisation Survey (EBASE).

**Results:**

A total of 332 osteopaths completed the survey. The demographic characteristics of respondents were generally consistent with the characteristics of the Australian osteopathy workforce. The respondents were mostly favourable of EBP, with the majority agreeing or strongly agreeing that EBP assists in making decisions about patient care (86.7%) and improves the quality of patient care (75.6%). While most respondents (88.3%) had some training in EBP, most reported a moderate level of perceived skill in EBP. The majority of respondents engaged infrequently (0–5 times) in EBP activities within the last month, and most indicated that a very small or small proportion of their clinical practice was based on clinical research evidence. Leading barriers to the uptake of EBP were lack of time and lack of clinical evidence in osteopathy. Key enablers of EBP uptake were access to the internet and online databases at work, and access to full-text articles and EBP education materials.

**Conclusions:**

Osteopaths participating in the survey were largely supportive of evidence-based practice, yet engaged infrequently in EBP activities. An important next step in this research is to identify suitable strategies that effectively improve EBP uptake in osteopathy, and perchance, improve patient outcomes.

## Background

Osteopathy has been described as a person-centred approach to manual therapy that focuses on the neurological, musculoskeletal and visceral structures of the body [1]. Osteopaths typically utilise a range of therapeutic interventions (including manual therapy, exercise and lifestyle advice) to manage diverse health complaints, although neuro-musculoskeletal conditions are the predominant focus. In 2013, there were an estimated 130,850 osteopaths / osteopathic physicians practicing in 33 countries across the globe [1]. In Australia - where osteopaths are considered primary care professionals - there were 2,277 practicing registered osteopaths (in 2018) [2].

Osteopaths play an important role in the delivery of musculoskeletal healthcare. In fact, musculoskeletal back pain is the leading reason why patients consult an osteopath [1]. As for the level of service provision, findings from a recent survey of the Australian osteopathy workforce suggest that osteopaths manage approximately 3.9 million patients per year. With osteopaths working 28 clinical hours per week on average, this equates to an estimated 3 million hours per year of patient care [3].

Osteopathy has achieved some form of national statutory regulation in a number of countries across the world, including several European countries, the UK, New Zealand and Australia [4]. The development of osteopathy from its inception in the USA during the late 1800s, has taken different paths globally over the course of time. The most marked differences can be found between ‘osteopathic physicians’ and ‘osteopaths’.

Osteopathic physicians primarily work in the USA, and are licensed to practice the full scope of medicine, including surgery and the prescription of medications, but they rarely specialise in the use of manual therapy techniques in practice [5]. By contrast, osteopaths, practicing outside of the USA, focus on the diagnosis, treatment, prevention and rehabilitation of musculoskeletal disorders, and the effects of these conditions on a patients’ general health, using predominantly hands-on manual therapy skills [6–8]. It is worth noting that a range of professional views and identities reside within osteopathy [9–11], and there is continued debate across the profession globally as to the particular theoretical, philosophical, and evidential underpinnings that define osteopathy and guide clinical practice and reasoning [12–14].

The role that research evidence plays in informing osteopathy practice and clinical decision-making is another area that has been keenly discussed across the globe [15–19]. Developed in the early 1990s [20], evidence-based medicine has been ubiquitously defined as the conscientious, explicit and judicious use of current best evidence in making decisions about the care of individual patients [21]. From its conception, evidence-based medicine and subsequent iterations, including evidence-based practice (EBP), emphasised that research evidence should be integrated with a clinician’s expertise (i.e. proficiency, values and judgment) [21]. In more recent times, there has been an increasing emphasis on the role of the patient in EBP (by incorporating a patient’s individual values, preferences and experiences in a process of shared decision-making) [22, 23].

Recent research has highlighted several challenges with embedding EBP into osteopathic practice and education. For example, in a survey of 370 UK osteopaths, Weber and Rajendran [24] found that even though osteopaths had largely positive attitudes towards EBP, a perceived lack of time and an inability to apply research evidence to individual patients were perceived barriers to EBP uptake. Similarly, findings from qualitative research have highlighted tensions between traditional osteopathic theory and EBP amongst UK osteopaths [12]; whereby knowledge, theory and opinion gathered from prominent individual ‘experts’ throughout osteopathy’s development from the 1800s frequently took precedence over external research evidence when making clinical decisions [25, 26]. Further qualitative research involving Australian osteopaths has also identified a perceived fear among clinicians that EBP will diminish or undermine the application of traditional osteopathic theory that is perceived to be unique to the profession [10]. In the context of this current study, these findings are important as some professional groups in Australia, specifically GPs, perceive there to be a lack of research evidence supporting osteopathic care [27, 28]; as gatekeepers of secondary and tertiary health care, GP (and other health provider) perceptions may represent a legitimate barrier to patient referral for publicly-funded osteopathy services in Australia.

Our previous research examined the barriers and facilitators of EBP uptake amongst UK osteopaths [29]. The work identified lack of time and a paucity of clinical evidence in osteopathy as key barriers to EBP uptake; access to online databases, internet at work, full-text articles, and EBP education materials were perceived to be important enablers of EBP utilisation. Whether these findings apply to osteopaths in Australia is unknown. Therefore, the aim of the study described herein was to investigate Australian osteopaths’ attitudes, skills and utilisation of research evidence in practice, their training in EBP, as well as the barriers and enablers of EBP uptake.

## Methods

### Design

National, cross-sectional survey.

### Research questions

The study was designed to answer the following research questions:

1. To what extent do Australian osteopaths engage in evidence-based practice (EBP)?
2. What level of importance do Australian osteopaths place on EBP?
3. What factors enable Australian osteopaths to practice EBP?
4. What barriers prevent Australian osteopaths from practicing EBP?
5. What skills and level of training do Australian osteopaths possess in order to practice EBP?
6. What types of interventions would facilitate Australian osteopath uptake of EBP?
7. Is there an association between practitioner demographics, and EBP use, skill and attitude?

### Sample and setting

All osteopaths registered with the Osteopathy Board of Australia (OBA), and practicing osteopathy in any state or territory in Australia, were eligible to participate in the study. Based on a target population of 2,277 practicing registered osteopaths (as at 31st March 2018) [2], the study required at least 329 respondents in order to achieve a 5% margin of error with 95% confidence for any individual item on the survey.

### Measurement

Practitioner attitudes, perceived skill, training and utilisation of EBP, and the barriers and enablers of EBP uptake, were measured using the Evidence-Based practice Attitude and utilization Survey (EBASE). This instrument has been administered to diverse practitioner populations to date, including chiropractors [30–32], herbalists [33, 34], naturopaths [33], yoga therapists [35] and nursing students [36]. EBASE has also undergone psychometric evaluation, and has been shown to have good internal consistency, construct validity, content validity, and acceptable test-retest reliability [37, 38].

The 84-item EBASE instrument comprises seven parts, with each part measuring a different construct: Part A (attitude toward EBP), Part B (EBP-related skills), Part C (EBP-related training), Part D (use of EBP), Part E (barriers to EBP uptake), Part F (enablers of EBP uptake), and Part G (demographic characteristics). Three subscores can be generated from EBASE: an attitude subscore, skill subscore and use subscore. The scoring procedures and parameters of these subscores are reported in detail elsewhere [32].

As EBASE was originally written for a general complementary and alternative medicine (CAM) audience, some terminology had to be modified to ensure the survey was relevant to Australian osteopaths. Specifically, the term CAM was replaced with osteopathy, and the response options of two demographic questions were revised (i.e. types of treatment / management typically provided in the first consultation; professional association membership). These minor amendments did not change the meaning of the questions, and therefore, did not impact the validity or reliability of EBASE.

### Recruitment and data collection

Eligible participants were invited to participate in the survey via a range of digital recruitment strategies. Members of Australia’s two largest osteopathy professional associations (i.e. Chiropractic and Osteopathic College of Australia; Osteopathy Australia) and an osteopathy practice-based research network (i.e. Osteopathy Research and Innovation Network [ORION]) were posted an invitation by email, with a reminder email posted 2 weeks later. Links to the survey were also disseminated via posts on social media, including the research team’s Twitter, Instagram and LinkedIn accounts, as well as pertinent Facebook pages.

All recruitment materials provided a web-link to the subject information sheet and online survey, which was hosted by SurveyMonkey™ (SurveyMonkey Inc., San Mateo, California, USA [www.surveymonkey.com]). Participants providing informed consent to participate (i.e. declaring that they met the eligibility criteria, understood what participation in the study involved, and understood what their rights were as a participant), were able to commence the survey. All survey items were made compulsory in order to mitigate the risk of missing data. The estimated completion time of the survey was 10–15 min. Data collection was undertaken between March 2018 and May 2018.

### Data analysis

Survey data were imported into SPSS (v.25.0) for coding and statistical analysis. Surveys identified as being partially-complete (i.e. more than 20% of items were unanswered due to respondents dropping out of the survey) were excluded from the analysis [35]. Multiple responses from single participants were handled using the de-duplication method for online surveys as described by Konstan et al. [39]. All missing data were described as missing values. Frequency distributions and percentages were used to describe categorical data. For normally distributed descriptive data, means and standard deviations were used. For non-normally distributed descriptive data, medians and the interquartile range (IQR: which were reported as a range rather than a value) were used. Relationships between nominal-level variables were examined using Cramer’s V, and associations between ordinal-level variables assessed using Kendall’s Tau correlation coefficient (Ƭ). Coefficients between 0.10–0.29 represented a weak correlation, 0.30–0.49 a moderate correlation, and 0.50–1.00 a strong correlation. Variables included in all tests of association were informed by previous studies using EBASE [30–35], and determined a priori. The significance threshold was set at *p* < 0.05.

## Results

A total of 368 Australian osteopaths completed the survey. Excluding multiple responses from single respondents (*n* = 2) and surveys with more than 20% unanswered items (*n* = 34), the adjusted sample size was 332. This exceeded the minimum sample size required for the study. As the number of osteopaths receiving an invitation to participate could not be determined, it was not possible to report an exact survey response rate.

### Demographic characteristics

Survey respondents were predominantly female (51.8%), aged between 30 and 49 years (54.5%) (Table 1). Most (59.6%) held a Master’s degree qualification, with the greatest proportion of respondents (44%) receiving their highest qualification 11 or more years prior. Correspondingly, the majority (48.8%) of respondents had practiced in the field of osteopathy for 11 or more years, with most (66.2%) working 16–45 h a week in clinical practice. Few respondents participated in research (0 h/week, 47.9%) or teaching in the higher education sector (0 h/week, 71.1%).

**Table 1:** Demographic characteristics of sample (*n* = 332)

Characteristic	Frequency
Age, *n (%)*	
*20–29 years*	52 (15.7)
*30–39 years*	109 (32.8)
*40–49 years*	72 (21.7)
*50–59 years*	39 (11.7)
*60–69 years*	13 (3.9)
*70+ years*	2 (0.6)
*Missing*	45 (13.6)
Sex, *n (%)*	
*Female*	172 (51.8)
*Male*	113 (34.0)
*Other*	2 (0.6)
*Missing*	45 (13.6)
Highest qualification, *n (%)*	
*Diploma/Advanced Diploma*	11 (3.3)
*Bachelor degree*	50 (15.1)
*Honours degree*	11 (3.3)
*Graduate Certificate/Diploma*	12 (3.6)
*Master’s degree*	198 (59.6)
*PhD/Professional doctorate*	5 (1.5)
*Missing*	45 (13.6)
Years since receiving highest qualification, *n (%)*	
* < 1 year*	14 (4.2)
*1–5 years*	63 (19.0)
*6–10 years*	64 (19.3)
*11–15 years*	69 (20.8)
*16+ years*	77 (23.2)
*Missing*	45 (13.6)
Years practiced in the field of osteopathy, *n (%)*	
*< 1 year*	10 (3.0)
*1–5 years*	49 (14.8)
*6–10 years*	66 (19.9)
*11–15 years*	64 (19.3)
*16+ years*	98 (29.5)
*Missing*	45 (13.6)
Hours per week in clinical (osteopathic) practice, *n (%)*	
*0 h*	2 (0.6)
*1–15 h*	43 (13.0)
*16–30 h*	108 (32.5)
*31–45 h*	112 (33.7)
*46+ hours*	22 (6.6)
*Missing*	45 (13.6)
Hours per week participating in research, *n (%)*	
*0 h*	159 (47.9)
*1–15 h*	121 (36.5)
*16–30 h*	4 (1.2)
*31–45 h*	1 (0.3)
*46+ hours*	1 (0.3)
*Missing*	46 (13.9)
Hours per week teaching in the higher education sector, *n (%)*	
*0 h*	236 (71.1)
*1–15 h*	41 (12.4)
*16–30 h*	8 (2.4)
*31–45 h*	1 (0.3)
*46+ hours*	0 (0.0)
*Missing*	46 (13.9)
Treatments typically provided in first osteopathic consultation, *n (%)*	
*Articulation*	263 (79.2)
*Soft tissue therapy*	246 (74.1)
*Muscle energy therapy*	240 (72.3)
*HVLA thrust*	212 (63.9)
*Exercise*	209 (63.0)
*General osteopathic treatment*	185 (55.7)
*Myofascial release*	181 (54.5)
*Strain-counterstrain*	160 (48.2)
*Functional technique*	112 (33.7)
*Relaxation advice*	104 (31.3)
*Cranial technique*	77 (23.2)
*Other*	70 (21.1)
*Visceral therapy*	57 (17.2)
*Acupuncture/acupressure*	26 (7.8)
*Ice/cold treatment*	24 (7.2)
*Orthotics*	6 (1.8)
*Electrotherapy*	5 (1.5)
*Steroid injection*	1 (0.3)
Clinical setting in which osteopathy was predominantly practiced, *n (%)*	
*With a group of CAM providers*	102 (30.7)
*Solo practice*	76 (22.9)
*With a group of conventional providers*	69 (20.8)
*With CAM & conventional providers*	34 (10.2)
*Within an educational institution*	3 (0.9)
*Missing*	48 (14.5)
Geographical location*, n (%)*	
*Victoria*	151 (45.5)
*New South Wales*	78 (23.5)
*Queensland*	27 (8.1)
*Tasmania*	13 (3.9)
*Western Australia*	7 (2.1)
*South Australia*	5 (1.5)
*Australian Capital Territory*	2 (0.6)
*Northern Territory*	1 (0.3)
*Missing*	48 (14.5)
Osteopathy professional association membership, *n (%)*	
*Osteopathy Australia*	268 (80.7)
*Not a member of an Osteopathy professional association*	9 (2.7)
*Chiropractic and Osteopathic College of Australasia*	4 (1.2)
*Other*	2 (0.6)
*Missing*	49 (14.8)
Geographical region, *n (%)*	
*Inner city suburbs*	107 (32.2)
*Outer city suburbs*	88 (26.5)
*Rural/remote region*	58 (17.5)
*City (Central business district)*	26 (7.8)
*Missing*	53 (16.0)

*CAM* Complementary and alternative medicine, *HVLA* high-velocity low amplitude

Respondents worked in various clinical practice settings, with a slightly higher proportion (30.7%) working in clinics with other CAM providers (Table 1). These practices were largely located in inner/outer city suburbs (58.7%) within the Australian state of Victoria (45.5%). The treatments typically provided by respondents in their first consultation with patients were diverse, with most using articulation (79.2%), soft tissue therapy (74.1%), and muscle energy therapy (72.3%).

### Attitude toward EBP

Respondents reported a median attitude subscore of 31 (IQR 27, 34; range 15–40), suggesting attitudes toward EBP were generally positive (with scores between 24.1 and 31.9 indicative of a predominantly neutral to agree response). In particular, respondents largely agreed that professional literature and research findings are useful for practice (83.4%), and that EBP assists in clinical decision making (86.7%), is necessary in the practice of osteopathy (84.6%), improves the quality of patient care (75.6%), and is fundamental to the advancement of the profession (73.2%) (Table 2). The majority of respondents were also interested in learning or improving the skills necessary to incorporate EBP into practice, with 87.6% agreeing or strongly agreeing to this. By contrast, many respondents disagreed/strongly disagreed that the adoption of EBP places an unreasonable demand on practice (59.9%).

**Table 2:** Respondent attitudes toward evidence-based practice (*n* = 332)

	*1*	*2*	*3*	*4*	*5*	Median (IQR)
	Strongly Disagree *n (%)*	Disagree *n (%)*	Neutral *n (%)*	Agree *n (%)*	Strongly Agree *n (%)*	
Professional literature (i.e. journals & textbooks) and research findings are useful in my day-to-day practice	3 (0.9)	21 (6.3)	31 (9.3)	194 (58.4)	83 (25.0)	4 (4,5)
EBP assists me in making decisions about patient care	1 (0.3)	17 (5.1)	26 (7.8)	188 (56.6)	100 (30.1)	4 (4,5)
I am interested in learning or improving the skills necessary to incorporate EBP into my practice	1 (0.3)	13 (3.9)	27 (8.1)	168 (50.6)	123 (37.0)	4 (4,5)
EBP is necessary in the practice of osteopathy	4 (1.2)	14 (4.2)	33 (9.9)	164 (49.4)	117 (35.2)	4 (4,5)
EBP improves the quality of my patient’s care	2 (0.6)	30 (9.0)	49 (14.8)	151 (45.5)	100 (30.1)	4 (4,5)
There is a lack of evidence from clinical trials to support most of the treatments I use in my practice	10 (3.0)	64 (19.3)	74 (22.3)	142 (42.8)	42 (12.7)	4 (3,4)
Prioritizing EBP within osteopathic practice is fundamental to the advancement of the profession	9 (2.7)	32 (9.6)	48 (14.5)	141 (42.5)	102 (30.7)	4 (3,5)
EBP takes into account my clinical experience when making clinical decisions	12 (3.6)	83 (25.0)	64 (19.3)	115 (34.6)	58 (17.5)	4 (2,4)
EBP takes into account a patient’s preference for treatment	21 (6.3)	110 (33.1)	81 (24.4)	90 (27.1)	30 (9.0)	3 (2,4)
The adoption of EBP places an unreasonable demand on my practice	36 (10.8)	163 (49.1)	76 (22.9)	49 (14.8)	8 (2.4)	2 (2,3)

*EBP* Evidence-based practice, *IQR* Interquartile range

A weak negative association was observed between attitude subscore (categorised by quartiles) and years since receiving highest qualification (Ƭ = -0.128, *p* = 0.012). A weak positive association between attitude subscore and hours per week participating in research (Ƭ = 0.164, *p* = 0.003) was also found. Associations between attitude subscore and other demographic characteristics were not shown to be statistically significant.

### Skills in EBP

Respondents reported a median skills subscore of 40 (IQR 33, 46; range 15–65), signifying a mostly moderate level of perceived skill in EBP (with scores between 39.1 and 51.9 indicative of a predominantly moderate to somewhat high skill level). Relatively higher perceived skill levels were reported for items pertaining to the first stage of the EBP process (i.e. clinical problem identification). The lowest perceived skill levels were reported for items relating to advanced research activities, such as the conduct of systematic reviews and clinical research, with 72.9 and 83.7% of respondents, respectively, reporting low to low-moderate skill levels for these tasks (Table 3).

**Table 3:** Respondent self-reported skills in evidence-based practice (*n* = 332)

	*1*	*2*	*3*	*4*	*5*	Median (IQR)
	Low *n (%)*	Low-moderate *n (%)*	Moderate *n (%)*	Moderate-high *n (%)*	High *n (%)*	
Identifying precise clinical questions	8 (2.4)	29 (8.7)	127 (38.3)	131 (39.5)	37 (11.1)	4 (3,4)
Identifying knowledge gaps in practice	5 (1.5)	20 (6.0)	128 (38.6)	130 (39.2)	49 (14.8)	4 (3,4)
Locating professional literature	16 (4.8)	56 (16.9)	85 (25.6)	99 (29.8)	76 (22.9)	4 (3,4)
Online database searching	28 (8.4)	45 (13.6)	90 (27.1)	93 (28.0)	76 (22.9)	4 (3,4)
Retrieving evidence	26 (7.8)	52 (15.7)	102 (30.7)	94 (28.3)	58 (17.5)	3 (3,4)
Critical appraisal of evidence	26 (7.8)	54 (16.3)	109 (32.8)	109 (32.8)	34 (10.2)	3 (3,4)
Synthesis of research evidence	37 (11.1)	83 (25.0)	110 (33.1)	79 (23.8)	23 (6.9)	3 (2,4)
Applying research evidence to patient cases	16 (4.8)	40 (12.0)	115 (34.6)	128 (38.6)	33 (9.9)	3 (3,4)
Sharing evidence with colleagues	27 (8.1)	71 (21.4)	103 (31.0)	86 (25.9)	45 (13.6)	3 (2,4)
Using findings from clinical research	12 (3.6)	45 (13.6)	129 (38.9)	123 (37.0)	23 (6.9)	3 (3,4)
Using findings from systematic reviews	41 (12.3)	73 (22.0)	108 (32.5)	81 (24.4)	29 (8.7)	3 (2,4)
Conducting systematic reviews	151 (45.5)	91 (27.4)	64 (19.3)	17 (5.1)	9 (2.7)	2 (1,3)
Conducting clinical research	202 (60.8)	76 (22.9)	33 (9.9)	18 (5.4)	3 (0.9)	1 (1,2)

*IQR* Interquartile range

Skill subscore (categorised by quartiles) was found to be weakly positively associated with age (Ƭ = 0.151, *p* = 0.002), highest qualification (Ƭ = 0.120, *p* = 0.022), hours per week teaching in the higher education sector (Ƭ = 0.231, *p* < 0.001) and hours per week participating in research (Ƭ = 0.273, p < 0.001). Associations between skill subscore and other demographic characteristics were not found to be statistically significant.

### Utilisation of EBP

Respondents reported a median use subscore of 7 (IQR 5, 11; range 0–24), representing a moderately-low level of engagement in EBP activities (with scores between 6.1 and 12.0 indicative of a moderately-low level of use). The majority (49.7–71.1%) of respondents participated in EBP activities (i.e. the first six items) no more than five times in the previous month. Most respondents also engaged infrequently (i.e. 0–5 times in the previous month) with the lay literature (80.7%) or with colleagues/industry experts (66%) (Table 4).

**Table 4:** Participant use of evidence-based practice (i.e. number of times each activity was undertaken within the last month) (*n* = 332)

	*0*	*1*	*2*	*3*	*4*	Missing *n (%)*	Median (IQR)
	0 times *n (%)*	1–5 times *n (%)*	6–10 times *n (%)*	11–15 times *n (%)*	16+ times *n (%)*		
I have read/reviewed professional literature (i.e. professional journals & textbooks) related to my practice	53 (16.0)	159 (47.9)	49 (14.8)	25 (7.5)	33 (9.9)	13 (3.9)	1 (1,2)
I have read/reviewed clinical research findings related to my practice	66 (19.9)	170 (51.2)	42 (12.7)	14 (4.2)	27 (8.1)	13 (3.9)	1 (1,2)
I have used professional literature or research findings to assist my clinical decision-making	43 (13.0)	168 (50.6)	47 (14.2)	22 (6.6)	39 (11.7)	13 (3.9)	1 (1,2)
I have used an online database to search for practice related literature or research	112 (33.7)	124 (37.3)	31 (9.3)	21 (6.3)	27 (8.1)	17 (5.1)	1 (0,2)
I have used professional literature or research findings to change my clinical practice	85 (25.6)	177 (53.3)	27 (8.1)	14 (4.2)	16 (4.8)	13 (3.9)	1 (0,1)
I have used an online search engine to search for practice related literature or research	21 (6.3)	144 (43.4)	67 (20.2)	30 (9.0)	53 (16.0)	17 (5.1)	1 (1,3)
I have consulted a colleague or industry expert to assist my clinical decision-making	49 (14.8)	170 (51.2)	58 (17.5)	18 (5.4)	20 (6.0)	17 (5.1)	1 (1,2)
I have referred to magazines, layperson / self-help books, or non-government/non-education institution websites to assist my clinical decision-making	115 (34.6)	153 (46.1)	31 (9.3)	5 (1.5)	11 (3.3)	17 (5.1)	1 (0,1)

*IQR* Interquartile range

A weak positive association was observed between use subscore (categorised by quartiles) and highest qualification (Ƭ = 0.119, *p* = 0.022), hours per week teaching in the higher education sector (Ƭ = 0.194, *p* < 0.001) and hours per week participating in research (Ƭ = 0.250, p < 0.001). There was also a weak negative association between use subscore and years since receiving highest qualification (Ƭ = -0.112, *p* = 0.034). Associations between use subscore and other demographic characteristics were not shown to be statistically significant.

Most respondents indicated that a very small (1–25% of practice; 28.6%) or small (26–50% of practice, 28.6%) proportion of their clinical practice was based on clinical research evidence. Those reporting a moderate (51–75% of practice) or large (76–99%) proportion of their practice as being based on clinical research evidence represented 25.9 and 7.8% of respondents, respectively. Few respondents indicated that none (1.8%) or all of their practice (0.9%) was informed by evidence from clinical trials. Traditional knowledge was the highest ranked information source (median rank 3; IQR 1, 6) used by respondents to inform clinical decision making. This was followed by clinical practice guidelines (median rank 3; IQR 3, 6) and consultation with fellow practitioners or experts (median rank 4; IQR 3, 6) (Table 5).

**Table 5:** Sources of information used to inform clinical decision-making (ranked by most frequent to least frequently used source)^a^ (*n* = 332)

Information source	Median (IQR)
Traditional knowledge	3 (1,6)
Clinical practice guidelines	3 (2,6)
Consulting fellow practitioners or experts	4 (3,6)
Published clinical evidence (i.e. clinical trials)	5 (2,8)
Textbooks	5 (3,7)
Personal intuition	5 (3,8)
Patient preference	6 (4,8)
Personal preference	6 (4,8)
Trial and error	8 (6,9)
Published experimental/laboratory evidence	9 (6,10)

*IQR* Interquartile range

^a^Sources were ranked from 1 = most frequently used, to 10 = least frequently used

### Training in EBP

The majority of respondents had undertaken some degree of training in evidence-based practice/osteopathy (88.3%), evidence application (78.9%), critical thinking/analysis (76.8%), and clinical research (61.8%), and to a lesser extent, the conduct of systematic reviews and meta-analyses (53.9%). In most cases, this training was completed as a minor (27.4–32.2%) or major (9.6–29.5%) component of a study program.

### Barriers to and enablers of EBP uptake

Respondents reported few barriers to the uptake of EBP in osteopathy, with 11 of 13 listed factors (i.e. lack of resources, skills in the location/interpretation/appraisal/application of evidence, incentive, interest, relevance, colleague/industry support, patient preference) being perceived as either not a barrier, or only a minor barrier to EBP uptake. The only factors identified as ‘moderate’ or ‘major’ barriers to EBP uptake were lack of clinical evidence in osteopathy (59.9%), and lack of time (52.7%).

Most respondents indicated that the 10 listed enabling factors facilitated the uptake of EBP in osteopathy, albeit with varying levels of perceived usefulness. Enablers considered to be ‘very useful’ by most participants were improving access to the internet in the workplace (69.6%), online EBP education materials (63.6%), free online databases (62.3%), databases requiring licence fees (57.2%) and critical reviews of research evidence relating to osteopathy (50.6%), as well as the ability to download full-text articles (63.0%). Among enablers perceived to be ‘moderately to very useful’ were access to critically appraised topics relating to osteopathy (69.9%), critical appraisal tools (60.5%) and research rating tools (58.7%), and online tools that facilitate practitioner appraisal of the evidence (51.5%).

## Discussion

This study has revealed some important insights into Australian osteopaths’ attitudes, skills and utilisation of evidence-based practice, as well as the barriers and enablers of EBP uptake among this professional group. Australian osteopaths were generally supportive of EBP, but largely reported low levels of EBP uptake in clinical practice. Further, despite most respondents completing some form of EBP-related training, perceived EBP skill levels were generally modest. Understanding the implications of these and other identified barriers and enablers of evidence-based practice uptake is a logical next step of this research, and consequently, is the focus of this discussion.

### EBP skills

Encouragingly, respondents reported positive attitudes to the role of EBP in osteopathic practice. They also reported moderate-to-high levels of self-perceived skill in the identification of clinical questions and knowledge gaps in practice, as well as appraising and applying evidence from research to their clinical practice. These findings were similar to the perceived EBP skill level reported by UK osteopaths [29]. Interestingly, respondents in the current study judged their skills in the relatively high-level tasks of ‘critical appraisal’ and ‘evidence synthesis’ to be of similar level as the more fundamental skill of ‘using findings from systematic reviews’. Notwithstanding, it should be emphasised that these surveys reported self-perceived skill in EBP and did not measure the actual skill level of respondents. It is possible that respondents who lack comprehensive knowledge of EBP could have over-estimated their skill level [40].

The lowest perceived skill levels relating to EBP were reported for the conduct of clinical research and/or systematic reviews. Given that this survey was distributed to practicing osteopaths, most without academic or research affiliations, it should be expected that they use research findings rather than produce them. Again, our findings are similar to recent studies, where Malaysian physiotherapists [41], US chiropractors [32] and UK osteopaths [29] were found to be confident in information appraisal, but lacked research skills. The small percentage of osteopaths reporting high-level skill in conducting clinical research or systematic reviews was also similar to previous studies of US and Canadian chiropractors where less than 5 % of respondents reported a high level of skill in these areas [31, 32]. Although allied health professionals should not necessarily be responsible for conducting research or literature reviews [42], we would expect that osteopaths should be able to search for and apply findings from systematic reviews and evidence-based guidelines to their daily practice. This ability is unlikely to represent a major barrier to EBP uptake amongst Australian osteopaths, with respondents reporting moderate to moderate-high level skills in these areas.

### Use of EBP

Despite the favourable view of EBP and moderately-high level of perceived skills in EBP, the majority of respondents reported low levels of engagement in evidence-based practice activities (i.e. they participated in these activities no more than five times in the previous month). Surprisingly, over a quarter of respondents reported that they never used an online database to search for practice-related literature or used this literature to change clinical practice over the last month. Similarly, about 20% of respondents reported that they never read or reviewed clinical research findings related to practice in the previous month. Instead, only around 15 % of respondents regularly reviewed clinical research findings, searched online databases or used professional literature to assist in daily clinical decision-making. Adding to this, nearly 60% of respondents reported very small/small proportions of their clinical practice to be based on EBP.

Most respondents relied upon traditional knowledge, clinical practice guidelines and fellow professionals to inform their clinical decision making. What is not clear is whether this dependency on traditional knowledge in Australian osteopathic practice is largely a ‘capacity’ issue (i.e. insufficient scientific evidence in the field, lack of time or sufficient skill to engage in EBP), a more entrenched ‘cultural’ issue (i.e. widespread disinterest in research, perception that the effectiveness of osteopathy is not amenable to scientific testing), or a product of both of these factors, or something entirely different [43]. Regardless of the reason, keeping up-to-date with current research, not in place of but as a complement to other relevant strategies, must be considered an important aspect of clinical practice in contemporary osteopathy. Thus, strategies and initiatives to increase EBP engagement in osteopathy practice may be important topics for future research.

Although the findings of the current study, and those of our recent UK study [29] indicate that Australian and UK osteopaths engage in EBP activities to a similar extent, the level of engagement in EBP appears to be somewhat lower than other manual therapy professions. In analogous studies involving chiropractors in the US [32] and Canada [31] (both of which used EBASE), approximately one-third of chiropractors reported reviewing professional literature / clinical research findings related to their practice, and using online search engines to search for practice-related literature, more than 11 times in the previous month [32]. By contrast, less than one-fifth of Australian and UK osteopaths engaged in the same activities to the same extent [29].

A possible explanation for the relatively low frequency of EBP activity among Australian and UK osteopaths may relate to the presentation of patients with a consistent range of symptoms and disorders that do not require frequent searching of evidence. However, if that were the case, one might expect chiropractors, and possibly physical therapists, to see a similar patient population. Yet, both chiropractors [31, 32] and physical therapists [44] report relatively higher levels of engagement in EBP activities. In one study of US physical therapists, 66% of respondents reported consulting research material and 52% having used a medical database, four to ten times weekly to make clinical practice decisions [44]. While a heterogeneous patient population might represent a probable reason for the differences in EBP utilisation between osteopaths and other manual therapists, other factors are equally possible, including differences in the level of research/EBP training, culture and opportunities for engagement [45].

### Barriers to EBP uptake

Given the positive attitude to evidence-based practice, but the low level of EBP utilisation among respondents, an examination of the barriers to EBP uptake should be revealing. However, participants perceived 11 of the 13 listed barriers to EBP as being only a minor barrier or not a barrier to EBP at all. The only factors identified as moderate or major barriers to EBP uptake were lack of clinical evidence in osteopathy and lack of time.

It is true that there is a lack of osteopathy-specific clinical research for both common and uncommon conditions treated by osteopaths; and while clinical evidence for osteopathic manipulative therapy is now emerging, methodological rigour is often lacking [46]. However, there is still much research in related disciplinary areas (e.g. physiotherapy, occupational therapy, chiropractic) that may be used to inform osteopathy practice [47, 48]. Thus, it is possible that the perceived ‘lack of clinical evidence’ was identified as a barrier to EBP uptake due to a poor understanding of the nature and activities of EBP. This level of understanding may stem from insufficient training in EBP, with the majority of respondents reporting some training in evidence-based practice, which was typically undertaken as a minor component of a professional study program. Although there have been calls to improve the development of EBP skills in osteopathy programs [49], it is likely the training offered over the last two decades has been variable and inadequate - particularly programs delivered more than 10 years ago.

Lack of time is not only a major barrier to EBP uptake reported by osteopaths [29], but also by nurses [50, 51], physiotherapists [44, 52, 53], chiropractors [31, 32, 54, 55] and other clinicians [56, 57]. However, some academics have argued that time is merely an excuse for not changing practice, and that clinicians playing the ‘lack of time card’ simply do not value EBP [58]. The same academics argue that these clinicians instead need some buy-in [58]. Indeed, studies of practicing chiropractors in the US, Canada, Australia and the UK indicate that lack of incentive is a notable barrier to EBP uptake [31, 32, 54, 55]. What is not clear at this point in time however, is whether incentivisation effectively improves EBP uptake, which of course should be a focus of further enquiry.

### Enablers of EBP uptake

Australian osteopaths agreed that research findings are useful for practice, and that EBP assists in clinical decision making and is necessary in the practice of osteopathy. The majority of respondents also identified themselves as having a moderate or moderate-high level of EBP skills. Despite this, the reported frequency of EBP activity was low. In the absence of many identified barriers to EBP beyond relevant evidence and time constraints, the perceived enablers of EBP may provide useful insights on how to promote greater uptake of EBP in osteopathy.

Two factors perceived by respondents as being particularly helpful in enabling EBP uptake in osteopathy practice were accessibility of evidence (i.e. access to the internet, databases and full-text articles in the workplace) and access to EBP training (particularly online EBP education materials). These enabling strategies were consistent with those reported by UK osteopaths [29] and Canadian chiropractors [31]. However, internet connectivity in the workplace is now largely ubiquitous, and primary online medical databases, such as PubMed, PEDro and The Cochrane Library, are free to access (at least in Australia) and include many open access full-text articles. Further, 90% of registered Australian osteopaths are members of the main osteopathic professional body [59], which provides members with access to full-text articles through various databases and journals. It is therefore unlikely that further efforts to expand clinician access to online resources would greatly enhance EBP uptake. Instead, the Australian osteopathy profession should consider placing emphasis on continuing profession education in EBP as a more suitable approach to improve the adoption of EBP within the osteopathic workforce. At present, there are no requirements for registered osteopaths (at least in Australia) to undertake continuing education in EBP.

### Limitations

While it is not possible to accurately determine the response rate to this survey due to the nature of sampling/recruitment, the survey appeared to be completed by approximately 14.6% (332/2277) of Australian osteopaths. This not only exceeded the minimum sample size required for this study, but also the response rates for other EBP surveys involving complementary medicine disciplines, including Canadian chiropractors (8%) [31], UK osteopaths (7.2%) [29], US yoga therapists (7.1%) [35] and US chiropractors (2.2%) [32]. Notwithstanding, there are some limitations to this study that are worth noting. As with any survey examining attitudes, it is possible that participants with an interest in EBP might have been more likely to participate in this study, which may have inadvertently introduced some degree of selection bias. In the event that selection bias was present, it is probable that participant attitudes to EBP may be generally more positive than that reported across the osteopathy profession, and that the level of engagement in EBP activities may be less frequent. However, as the demographic profile of participants closely approximated the age, sex, geographical distribution, type of practice setting and highest qualification of Australian osteopaths, it is probable that the study sample was broadly representative of the Australian osteopathy workforce [2]. Other limitations inherent in the survey design include the reliance on self-reported information and recall bias. Additionally, perceived skill level can be tainted by cognitive bias, particularly among participants with low-level knowledge and skill, which may result in over-estimation of such knowledge and skill (referred to as the Dunning-Kruger effect) [40].

The above limitations, as well as the understandings gained from this study, highlight the need for further research in this field. For instance, there is a need to investigate the skill/competency level of osteopaths with regards to applying EBP, and to better understand the skills that are required for the successful integration of EBP into osteopathic practice. A related area of research is the development, implementation and evaluation of appropriate interventions that facilitate the uptake of EBP by the osteopathy workforce. Such work could be facilitated through improved collaboration between professional, educational and academic research bodies, as has already been demonstrated through the recent Osteopathy Research and Innovation Network (ORION) project [3].

## Conclusions

This study has revealed important insights into Australian osteopath attitudes, skills and utilisation of EBP. Overall, respondents were generally positive toward EBP, and the majority agreed or strongly agreed that EBP assists in making clinical decisions, improves the quality of patient care, and is necessary in the practice of osteopathy. Despite the majority of respondents reporting a moderate or moderately-high level of EBP skills, the level of engagement in EBP activities over the preceding month was low. Principle barriers to EBP were identified as lack of time and a paucity of clinical evidence in osteopathy. The main enablers of EBP uptake related to improving access to evidence and training in EBP. The findings suggest that continuing professional education initiatives in EBP may be valuable in assisting osteopaths to more frequently engage in EBP activities in clinical practice.

## Data Availability

The datasets used and/or analysed during the current study are available from the corresponding author on reasonable request.

## Abbreviations

CAM: Complementary and alternative medicine
EBASE: Evidence-Based practice Attitude and utilization SurvEy
EBP: Evidence-based practice
HVLA: High-velocity low amplitude
IQR: Interquartile range
